# Companied P16 genetic and protein status together providing useful information on the clinical outcome of urinary bladder cancer

**DOI:** 10.1097/MD.0000000000010353

**Published:** 2018-04-13

**Authors:** Xiaohong Pu, Liya Zhu, Yao Fu, Zhiwen Fan, Jinyu Zheng, Biao Zhang, Jun Yang, Wenyan Guan, Hongyan Wu, Qing Ye, Qing Huang

**Affiliations:** a Department of Pathology, Nanjing University Medical School affiliated Drum Tower Hospital, Nanjing; b Department of Blood Purification Center, Huan’an First People's Hospital, Nanjing Medical University, Jiangsu, China; c Department of Pathology and Laboratory Medicine, Veterans Affairs Boston Healthcare System and Harvard Medical School, West Roxbury, MA.

**Keywords:** FISH, immunocytotochemistry, P16 genetic status, p16^INK4a^ protein, urinary bladder cancer

## Abstract

SPEC P16/CEN3/7/17 Probe fluorescence-in-situ-hybridization (FISH) has become the most sensitive method in indentifying the urothelial tumors and loss of P16 has often been identified in low-grade urothelial lesions; however, little is known about the significations of other P16 genetic status (normal and amplification) in bladder cancer.

We detected P16 gene status by FISH in 259 urine samples and divided these samples into 3 groups: 1, normal P16; 2, loss of P16; and 3, amplified P16. Meanwhile, p16^INK4a^ protein expression was measured by immunocytochemistry and we characterized the clinicopathologic features of cases with P16 gene status.

Loss of P16 occurred in 26.2%, P16 amplification occurred in 41.3% and P16 gene normal occurred in 32.4% of all cases. P16 genetic status was significantly associated with tumor grade and primary tumor status (*P* = .008 and .017), but not with pathological tumor stage, overall survival, and p16 protein expression. However, P16 gene amplification accompanied protein high-expression has shorter overall survival compared with the overall patients (*P* = .023), and P16 gene loss accompanied loss of protein also had the tendency to predict bad prognosis (*P* = .067).

Studies show that the genetic status of P16 has a close relation with the stages of bladder cancer. Loss of P16 is associated with low-grade urothelial malignancy while amplified P16 donotes high-grade. Neither P16 gene status nor p16^INK4a^ protein expression alone is an independent predictor of urothelial bladder carcinoma, but combine gene and protein status together providing useful information on the clinical outcome of these patients.

## 1 Introduction

Urothelial carcinoma of the urinary bladder is one of the most common cancers in the world.^[1]^ The prognosis of patients with this carcinoma depends on pathologic stages, in which about 2% to 5% of cases are at pTis (carcinoma in situ), 40% at pT1a, 30% at pT1b, and 20% at pT2 or higher stages at diagnosis.^[1–4]^ Overall, 70% of treated tumors recur, among which 30% of recurrent tumors progress to metastatic disease from the initial nonmuscle-invasive lesions.^[2,4]^ Therefore, identification and establishment of a highly sensitive and specific method to predict carcinoma progression from noninvasive low-grade diseases to invasive and metastatic diseases becomes critically important for patient management.

At present, modern cystoscopy remains the “gold” standard for the detection of de novo and recurrent bladder cancer. Because the majority of patients may have a recurrence after endoscopic resection, a lifelong surveillance with cystoscopy is thus recommended.^[5]^ However, the high cost and invasive nature of cystoscopy limit its routine use, in addition to its frequent failure to accurately detect a flat neoplastic lesion, especially carcinoma in situ, which is a high grade malignant lesion with a poor prognosis.^[4]^ In contrast, as an “ancient“ technique, the urine cytology examination is increasingly used clinically to detect urothelial carcinoma because of its noninvasive nature, low cost, and high specificity. In the hands of a well-trained experienced cytopathologist, the specificity of urine cytology to detect urothelial carcinoma is greater than 90%,^[6]^ and the sensitivity for high-grade disease can be as high as 80% to 90%.^[7,8]^ However, the sensitivity of urine cytology for low-grade urothelial carcinoma is only 20% to 50%,^[4]^ which makes this old method less attractive to detect urothelial carcinoma at early stages. Although several biofactors and prognostic score system have been reported for early diagnosis and prognosis evaluation, large prospective trials are still needed to evaluate the reliability to predict tumor behavior.^[9–12]^ At present, urothelial carcinomas are known to have many chromosomal abnormalities, especially chromosomes 1, 3, 4, 7, 8, 9, 11, and 17,^[13–15]^ which can be detected by fluorescence-in-situ-hybridization (FISH) using DNA probes to chromosome centromeres or unique loci that are altered in tumor cells. For example, the UroVysion test detects gains in chromosomes 3, 7, and 17, and losses in chromosome 9p21 (p16 locus) with a higher sensitivity (90% by FISH vs 30% by cytology), but similar specificity (approaching 90%), compared to cytology for the diagnosis of urothelial carcinoma.^[16–20]^ p16, also known as cyclin-dependent kinase inhibitor 4A (CDKN4A), is a tumor suppressor gene that controls cell cycle progression.^[21,22]^ It is known that about 40% of urothelial carcinomas have a heterozygous loss of p16, which is a common carcinogenic mechanism involved in low-grade urothelial carcinomas.^[23]^ Loss of one or both alleles in the p16 gene is imperative for the transition of normal urothelium to papillary urothelial carcinoma.^[24,25]^ On the other hand, overexpression of the p16^INK4a^ protein is found in 11% to 100% of urothelial carcinomas,^[26]^ and also reported to be correlated with disease recurrence and progression.^[27]^ However, little is known about the significance of normal and amplified p16 gene in the clinical diagnosis of urothelial carcinoma of the urinary bladder. In this study, we collected 259 cases of urothelial carcinomas, compared the changes in expression of p16 gene and its protein levels in urine samples, and evaluated the predictive value of p16 gene statusin urothelial carcinoma of the urinary bladder.

## 2 Materials and methods

### 2.1 Patients and clinicopathological data

Between 2006 and 2015, urine samples were collected from 1050 patients who had clinical symptom and signs, such as gross or microscopic hematuria, and thus were suspected to have bladder cancer. All patients were tested by urine cytology and the FISH test for the presence or absence of urothelial carcinoma of the urinary bladder. As a result, 294 patients were diagnosed as bladder urothelial carcinoma after an initial workup procedure. They were recommended to have their tumors resected. Exclusion criteria for this study included: preoperative local or systematic anticancer neoadjuvant therapy (n = 8); incomplete clinical and pathological data (n = 17); no transurethral resection or radical cystectomy at our center (n = 5); and no information on immunocytotochemistry or FISH test results in tumor samples (n = 5). Thus, a total of 259 cases were eligible for this study. All patients were followed up for postresection outcomes. There was no significant difference in the prognostic role between transurethral resection and radical cystectomy.

Based on the 2010 World Health Organization (WHO) urothelial carcinoma diagnostic criteria and the 7th edition of the American Joint Committee on Cancer/International Union against Cancer TNM staging system of tumors of the urinary system (AJCC7),^[28]^ each resection specimen was reviewed with all available histologic slides by study pathologists to confirm the diagnosis, who also analyzed the morphologic features of bladder urothelial carcinoma. Tabulated was the information on patient age, gender, tumor maximum dimension, and tumor TNM staging. Follow-up was carried out every 6 months after discharge with cystoscopy, urinary cytology, and FISH testing chromosomes 3,7,17 and 9p21, or every 12 months with contrast-enhanced computerized tomography scan. Among 259 patients, 184 (71.0%, 184/295) had the complete follow-up information and 75 (29.0%, 75/259) were lost. The study protocol was approved by the Medical Ethics Committee of the Nanjing Drum Tower Hospital.

### 2.2 FISH analysis

According to the manufactural test protocol (Anbiping, Guangzhou China), at least 200 mL of first urine in the morning was collected using vials for the FISH test and the procedure was completed within 1 hour. After pretreatment with the phosphate buffered solution mixed with 0.5 mg/mL pepsin at 37 °C, 1% neutral buffered formaldehyde fixation, the cytology slides were dehydrated for 5 minutes each in ethanol from 70%, 80% to 100%. The FISH probe with p16 labeled on chromosome 9p21/9q21 (Anbiping, Guangzhou, China) was used. Codenaturation (5 minutes at 73 °C) and hybridization at 37 °C were carried out overnight in a hybridizer (DAKO, Carpinteria, CA). The procedure was followed by a postwash with 0.4× and then 2× saline sodium citrate. Diamidinophenylindole II was used as a counterstain. Appropriate positive and negative controls were carried out in each run. To analyze the FISH signals, the FISH labeled slides were scored in a dark room for hybridization signals under the Olympus BX 51 (Olympus, Japan) microscope equipped with a filter set for diamidinophenylindole (counterstain), red (p16, 9p21 locus), and green (9q21) band-passes.

Urothelial cells had no or only 1 red signal but 2 green signals were defined as p16 gene loss. Cells showed more than 3 red signals and 2 green signals, or the ratio of red/green signals more than 1.5 were diagnosed as p16 gene amplification. Cells containing 2 red and 2 green signals were considered p16 gene normal expression. In each sample, 100 urinary cells were counted in random. The p16 gene loss group was composed of more than 20% cells with p16 gene loss. Similarly, the p16 gene amplification group showed more than 20% cells with p16 gene amplification. The samples that did not meet the criteria for p16 gene loss or amplification were included in the p16 gene normal expression group.

### 2.3 p16^INK4a^ immunocytochemistry

At least 200 mL first urine in the morning were centrifuged at 1500 rpm for 5 minutes and fixed in 4% paraformaldehyde overnight. After cytological evaluation, the slides were immunostained for p16^INK4a^ protein expression, using a mouse monoclonal antibody to p16^INK4a^ (clone 16P04, ZhongshanJinqiao, Beijing, China) at a dilution of 1:100 with a standard immunocytochemistry protocol. Briefly, slides were immersed first in 1 mmol/L of EDTA at pH 9.0 for 30 minutes in a preheated vegetable steamer at 92 °C. Then the slides were allowed to cool down at room temperature for 5 minutes and rinsed with deionized water for 3 minutes. Subsequently, the slides were placed on a Dako Autostainer (DakoCytomation, Carpentaria, CA) and incubated with the p16 antibody for 30 minutes. The antigen-antibody reaction products were visualized using a dextran polymer-based detection system, ENVISION+ (DakoCytomation), and 3,3V-diaminobenzidine (DakoCytomation) as the substrate chromogen. Both positive and negative controls were included in each run to ensure the staining procedure validity, as required by the standard protocol.

**Figure 1:**
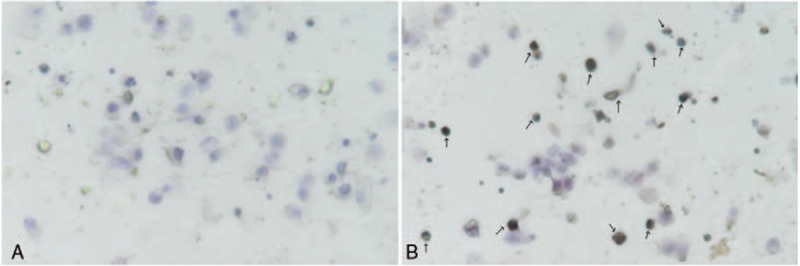
p16^INK4a^ immunohistochemistry staining negative cells (A) and p16^INK4a^ immunohistochemistry staining positive cells as the arrow points (B).

Each slide was examined to determine the total urothelial cell count and the number of P16 positive cells, nuclear or cytoplasmic staining intensity was considered positive (Fig. 1). A semiquantitative total urothelial cell count was performed at random area of the monolayer of 1000 urothelial cells. p16^INK4a^ immunohistochemistry staining was classified as 0 (negative, loss of p16^INK4a^ expression), 1+ (less than 100 urothelial cells with P16 positive), 2+ (positive staining of P16 between 100 and 300 urothelial cells), and 3+ (more than 300 urothelial cells with P16 positive).

In each test run, uterine cervical epithelial cells with proven p16^INK4a^ overexpression served as the positive control, and chronic inflammatory cells served as negative internal controls. The p16^INK4a^ immunostaining was evaluated and scored independently by an investigator who had no prior knowledge of the FISH test findings.

### 2.4 Statistical analysis

Distributed data of continuous variables were represented as “mean ± standard deviation (SD)” and range. Analysis of variance or the Kruskal–Wallis rank sum test was used to compare differences among 3 groups. The Chi-square or Fisher exact test was utilized for comparison of ratios. Patient postresection survival was estimated by the Kaplan–Meier method with a log rank test. Statistical analysis was performed with SPSS 19.0 software (SPSS Inc., Chicago, IL). Differences were considered to be statistically significant when *P* values were less than .05.

## 3 Results

As shown in Table 1, p16 gene expression was observed in 26.2% (68/259) of cases, normal P16 gene in 32.4% (84/259) of the cases, and amplification of P16 gene in 42.7% (107/259) of all the samples. Significant differences of tumor grade and primary tumor status evaluation within the 3 groups (*P* = .008 and .017). The results indicated different P16 genetic status according to different stages of histology, deletion of P16 indicates low grade, amplified P16 indicates high-grade, whereas normal P16 fits between them. As to the primary tumor status, P16 gene deletion group has less samples in Ta and more samples in T_3a_–T_4b_ than the P16 gene amplification group. No significant relationship between P16 gene status and other clinicopathological variables like age (*P* = .899), sex (*P* = .287), tumor size (*P* = .354), lymph node status (*P* = .522), lymphovascular status (*P* = .264), distant metastasis (*P* = .707), and stage (*P* = .116) were found.

**Table 1:**
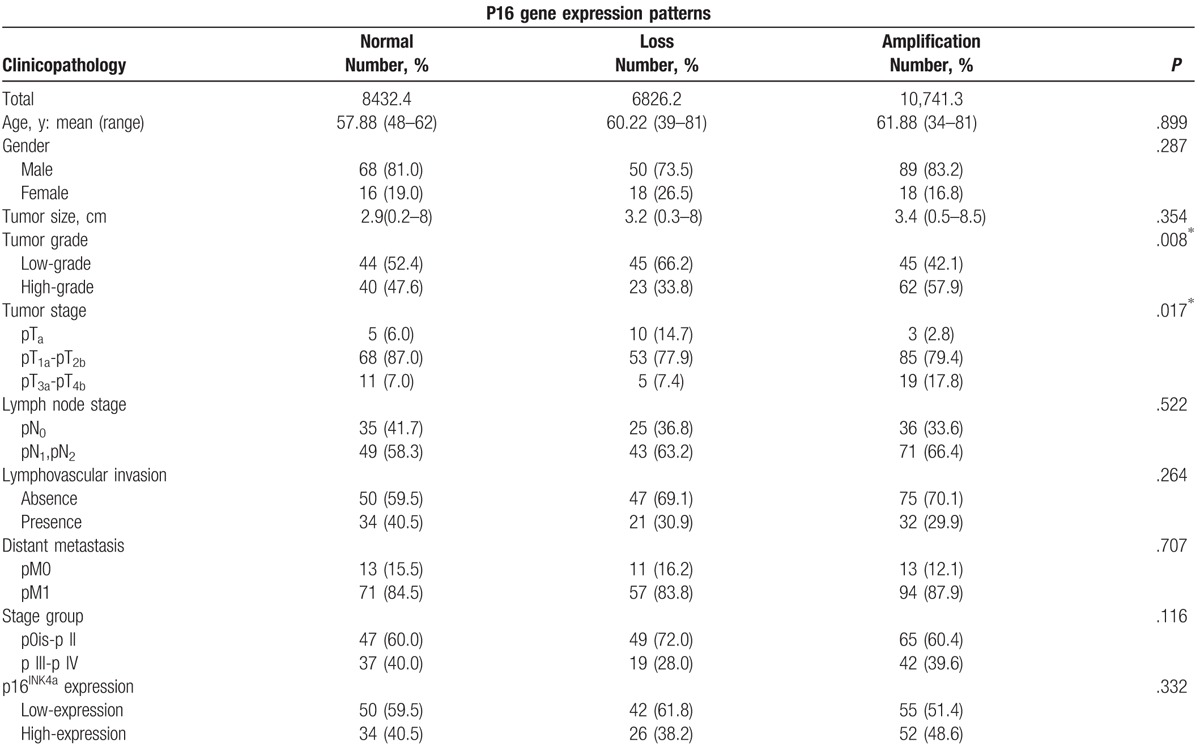
Comparison of clinicopathological characteristics among 3 groups of bladder urothelial carcinoma with different p16 gene expression patterns revealed by fluorescence-in-situ-hybridization (FISH) test.

**Figure 2:**
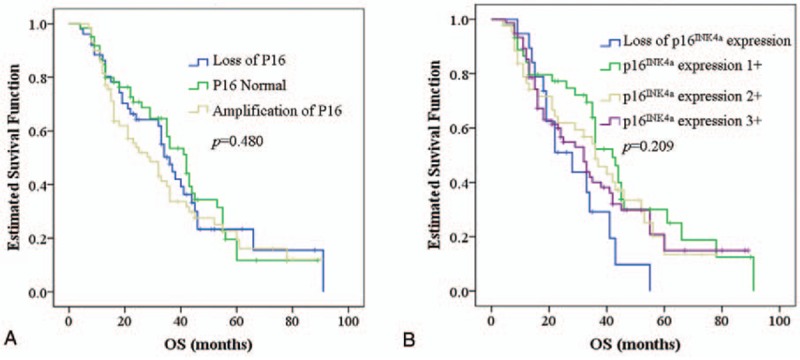
Kaplan–Meier overall survival analysis of different P16 genetic status (A) and protein status (B).

Of 259 cases included in the study, the overall survival was observed in 184 (71%) patients. An according Kaplan–Meier survival curve of the 3 different P16 gene patients was illustrated in Fig. 2A and showed no significant prognostic impact (*P* = .480). Moreover, p16^INK4a^ expression has no relation with clinicopathological classification (data not show) and cannot be identified as an independent predictor of prognosis as the survival curve of 4 different p16^INK4a^ expression groups were illustrated in Fig. 2B and showed no significant difference (*P* = .209).

**Figure 3:**
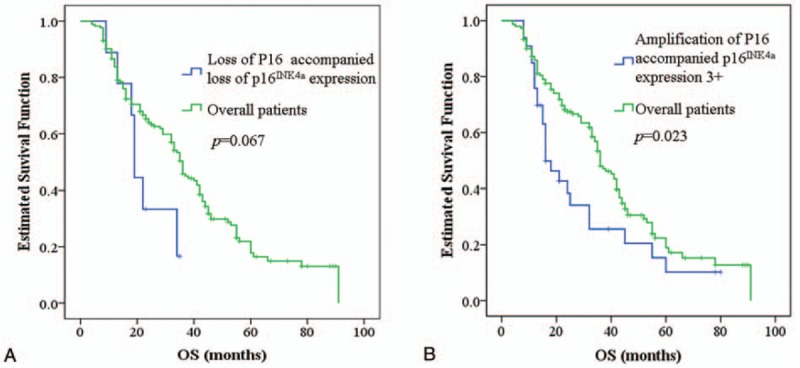
Kaplan–Meier overall survival analysis of P16 gene loss companied protein loss (A) and P16 gene amplification companied protein overexpression (B).

Table 2 shows p16^INK4a^ expression in urine cytologic samples. When we divided the low-expression in to 4 levels as loss of p16^INK4a^ expression (0), 1+, 2+, and 3+, there still yielded no significant correlation between immunocytochemical p16 status and P16 gene status. Although there was no significant difference (*P* = .332) in p16^INK4a^ expression (high-expression and low-expression) between the 3 different P16 gene group in urothelial carcinoma (Table 1), there were more samples in loss of p16^INK4a^ expression from loss of P16 gene loss group and p16^INK4a^ overexpression from P16 amplification group. Comparison of the overall survival of loss of P16 accompanied loss of p16^INK4a^ expression group and P16 gene amplification accompanied p16^INK4a^ overexpression group with the overall patients, these 2 variables showed significant predictive value (Fig. 3A and B). P16 gene amplification accompanied p16^INK4a^ overexpression group predicts a bad prognosis (*P* = .023), and loss of P16 accompanied loss of p16^INK4a^ expression group has the tendency (*P* = .067) to predict a bad prognosis of urinary bladder cancer.

**Table 2:**
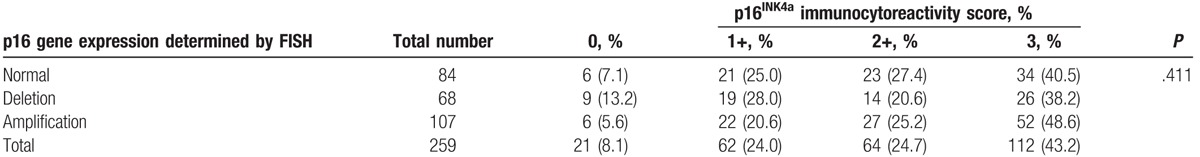
Correlation between the fluorescence-in-situ-hybridization (FISH)-determined p16 gene expression and p16^INK4a^ immunoreactivity in urothelial carcinomas of urine specimens.

## 4 Discussion

Aberrant p16 gene expression is known to affect carcinogenesis and progression of urothelial carcinoma,^[25,29,30]^ as demonstrated in this study. We showed that abnormal p16 gene expression in bladder urothelial carcinomas correlated with the tumor grade and confirmed the results of other studies for a significant association between loss of p16 gene expression and urothelial carcinomas.^[24,25]^ As reported previously,^[31]^ p16 gene has a negative regulatory effect on bladder carcinogenesis at the early stage. However, there are some controversies in the current literature about the relationship between p16^INK4a^ expression and prognosis.^[32,33]^ Some authors regarded that low expression of p16 gene was correlated with poor progression-free survival and recurrence free survival in early-stage (Tis–T1) bladder carcinomas, suggesting an important role in the development of bladder carcinoma.^[31,34]^ Others reported a high frequency of p16 overexpression in high-grade urothelial carcinomas as an indication for high tumor infiltrating potential.^[26]^ In fact, p16^INK4a^ expression has been proposed as a reliable biomarker for urothelial carcinoma in urine cytology samples,^[26,35]^ as demonstrated in our study. However, different from previous studies on correlation between loss of p16 gene expression and tumor progression,^[36,37]^ and p16^INK4a^ overexpression in different grades of urothelial carcinomas,^[33,37–39]^ our study showed no significant relationship between p16^INK4a^ expression and the patient survival. The apparent discrepancy may be attributable to the differences in the study design, sample type, and diagnostic criteria. For instance, p16 immunoreactivity was scored on a scale of 0 to 3 in some studies,^[40]^ as we did, while others interpreted p16 gene expression only as either negative or positive,^[38]^ or even simply as either normal or abnormal. In the latter scenario, both loss of p16^INK4a^ expression and p16^INK4a^ overexpression were classified as abnormal, whereas moderate p16^INK4a^ immunostaining was considered as normal.^[37,41–43]^ Although p16^INK4a^ is a marker expressed in both nuclei and cytoplasm,^[42]^ only nuclear p16 immunostaining was considered positive by some investigators.^[38,43,44]^ The inconsistency of interpretation of p16 test data resulted in considerable difficulty reconciling research results from different groups. Nevertheless, our data combined both p16^INK4a^ immunostaining and the p16 FISH quantitative results and demonstrated a clinically very useful scheme for detection of urothelial carcinoma of the bladder by p16 gene expression in urine cytology specimens. Our study results showed that loss of p16 gene expression was associated with low-grade urothelial carcinoma, while p16 gene amplification suggested the presence of high-grade urothelial carcinoma; importantly, a poor prognosis prodictof urothelial carcinoma in the urine specimen requires both positive p16^INK4a^ protein expression and a high score of the p16 FISH test in the same specimen. Our data on p16 normal and amplified expression in urothelial carcinoma of the urine specimen have not been reported previously. We found that a combination of both p16 gene status and its protein levels may help predict postresection prognosis. Nevertheless, our data only partially confirmed the prognostic relevance of a double loss of p16 gene and its protein expression with poor outcomes, because of the small sample size of only 9 patients with the loss of both p16 gene expression determined by the FISH test and its protein levels shown with immunocytochemistry, leading to a *P* value of .067.

In our study, the p16 immunoreactivity was not in accordance with the p16 FISH test results. There are several explanations for this inconsistency. First, the FISH 9p21 probe used in this study spans the p16 gene, but the illustration of the p16 locus at 9p21 by the FISH test would not be exactly expected to reflect the entire p16 gene status. Second, increased p16^INK4a^ expression by immunocytochemistry may be related to polyploidy of chromosome 9 or amplification of the 9p21 locus, which directly augments p16 gene expression at the protein level. Finally, p16 gene dysfunction on self-regulation, such as abnormally high levels of cell proliferation, may cause a very long half-life of p16^INK4a^ protein accumulated in cells, leading to strong immunoreactivity, but not the FISH detected p16 gene amplification.^[45]^

Most previous reports studied p16 gene expression in tumor resection tissue samples, but few used the urinary cytological samples.^[35]^ Urine specimens have many advantages for the detection of urothelial carcinoma and surveillance of recurrent carcinoma after resection. This is especially important for detection of urothelial carcinoma at its early stages. This is especially appealing for early detection of urothelial carcinoma at its early stages or early recurrence.

Recently, several biofactors have been reported as potential markers for early diagnosis and prognosis such as long noncoding RNA^[9,11]^ and circulating tumor cells.^[46]^ It is undeniable that these markers have brought revolution for the diagnosis and therapy strategy. Patients with the same histology characteristic may under individualized therapy based on these biomarkers. However, the markers still in the research and the methodologic efficacy have not yet been fully demonstrated until now. Nevertheless, both FISH and IHC detected the genetic and protein status of P16 in our study have been used for many years and demonstrated as the most stable, reliable, and sensitive methods. And we admit that, as other biomarkers, large prospective trials are needed to better evaluate how these markers could reliably predict tumor behavior together with the ability to guide target therapies.

The major limitation of this study included a small number of cases with both loss of p16 gene expression and immunoreactivity so that the difference did not reach a statistically significant level. At present, we are continuing the current clinical research project and collecting more such cases. Another shortcoming was related to a short length of follow-up. We are hoping as the project continues and the postresection outcome data would be improved.

In conclusions, we demonstrated in this study a new p16 gene expression biomarker that combined both the P16 gene amplification by FISH and p16^INK4a^ protein overexpression by immunocytochemistry to predict and diagnose urothelial carcinoma in urine cytology specimens.

Still, further studies are needed to support these new prognostic parameters.

## Acknowledgments

Foundation item: Six Talent Peaks Project in Jiangsu Province (WSW-073), Health Young Talent Training Project in Nanjing (QRX-17055), Innovation Capability Development Project of Jiangsu Province (No. BM2015004), Nanjing Health and Family Planning Commission medical science technology innovation platform project (ZDX16006), National Human Genetic Resources Sharing Service Platform (2005DKA21300), Key research and development Programs social development project of Science and Technology Commission Foundation of Jiangsu Province (BE2016604)

## Author contributions

**Data curation:** Xiaohong Pu, Zhiwen Fan.

**Funding acquisition:** Qing Ye.

**Investigation:** Jun Yang, Xiaohong Pu.

**Methodology:** Biao Zhang, Hongyan Wu, Liya Zhu, Wenyan Guan, Xiaohong Pu, Yao Fu.

**Validation:** Jinyu Zheng.

**Writing – original draft:** Xiaohong Pu.

**Writing – review & editing:** Qing Huang, Xiaohong Pu.
